# Effects of nasal dilator strips on subjective measures of sleep in subjects with chronic nocturnal nasal congestion: a randomized, placebo-controlled trial

**DOI:** 10.1186/s13223-018-0258-5

**Published:** 2018-08-27

**Authors:** Eric J. Schenkel, Renee Ciesla, Gilbert Marava Shanga

**Affiliations:** 1 Valley Clinical Research Center, 3101 Emrick Boulevard, Suite 211, Bethlehem, PA 18020 USA; 2 GlaxoSmithKline Consumer Healthcare, Warren, NJ USA

**Keywords:** Sleep, Nasal dilator strips, Breathe Right, Nasal congestion, Patient reported outcome measures

## Abstract

**Background:**

This exploratory study investigated effects of a new asymmetric butterfly-shaped prototype nasal dilator strip and the currently marketed clear Breathe Right Nasal Strip (BRNS) on subjective measures of nasal congestion and sleep quality.

**Methods:**

In this randomized, double-blind study, subjects with chronic nasal congestion and sleep difficulties were assigned a BRNS clear strip, an asymmetric butterfly prototype, or an asymmetric butterfly placebo strip without springs, to use nightly for 2 weeks. The main outcomes included change from baseline to days 7 and 14 on the Pittsburgh Insomnia Rating Scale (PIRS), Nocturnal Rhinoconjunctivitis Quality of Life Questionnaire (NRQLQ), and Congestion Quantifier Seven-Item Test (CQ7).

**Results:**

The intent-to-treat population included 59 subjects. The butterfly and BRNS strips showed significant (*P *< 0.05) improvement versus placebo on PIRS satisfaction with sleep at day 7 [least square (LS) mean changes: − 0.7, − 0.6, and − 0.2, respectively], and the butterfly strip also showed significant improvement from baseline on this outcome versus placebo at day 14 (− 1.0 vs − 0.5). On the NRQLQ, both the butterfly prototype and BRNS clear were more effective than placebo in improving symptoms on waking at day 7 (LS mean changes: − 7.9, − 7.2, and − 4.1, respectively); the BRNS clear was significantly more effective than placebo in improving sleep problems at day 7 (− 7.4 vs − 4.2). There were no between-treatment differences on the CQ7. All strips were well tolerated.

**Conclusions:**

The asymmetric butterfly prototype and BRNS clear strip significantly improved some subjective measures of nasal congestion and sleep compared with placebo in subjects with nasal congestion and sleep difficulties.

*Trial registration* This study is registered at ClinicalTrials.gov (identifier: NCT01122849)

**Electronic supplementary material:**

The online version of this article (10.1186/s13223-018-0258-5) contains supplementary material, which is available to authorized users.

## Background

Nasal congestion, which increases when lying down [1], is a predictor of moderate sleep difficulties [2]. Relief of nasal congestion (e.g., with topical nasal corticosteroids) has been associated with improvements in subjective, patient-reported sleep outcomes and a reduction in daytime sleepiness [3–6].

The Breathe Right^®^ Nasal Strip (BRNS; GlaxoSmithKline Consumer Healthcare; Parsippany, NJ, USA) is a nonpharmacologic adhesive nasal dilator strip containing two springs that is applied to the bridge of the nose [7–9]. By pulling outward on the nasal vestibule, the BRNS increases the cross-sectional area of the nasal valve region and the volume inside the anterior part of the nose, thereby opening the nasal passages [7–10] and reducing nasal airflow resistance [11]. The BRNS rapidly improves inspiratory nasal airflow and relieves subjective feelings of nasal congestion and obstruction [7, 8, 10, 12]. Use of BRNS during sleep has been found to improve sleep quality [13–15] and reduce snoring [13, 14, 16, 17], insomnia [15], and daytime sleepiness [13, 16].

An asymmetric butterfly prototype nasal dilator strip has been developed. The butterfly strip adheres to the cheek instead of the nose flare. This design was expected to pull outward on multiple areas of the nose compared with the BRNS, which pulls in a straight line across the nose. This exploratory study was designed to investigate whether nasal dilation with the new prototype strip and with the BRNS clear strip would have a positive effect on subjective, patient-reported outcomes of nasal congestion and sleep quality compared with placebo in subjects with chronic nasal congestion who reported sleep difficulties.

## Methods

### Study design and procedures

This was a randomized, placebo- and active-controlled, parallel-group, exploratory phase 2 study conducted at Valley Clinical Research Center, Bethlehem, PA, and TKL Research, Paramus, NJ from October 13, 2009 to February 4, 2010 (ClinicalTrials.gov identifier: NCT01122849). Subjects were randomly assigned to receive 1 of 3 treatments: a BRNS clear strip, an asymmetric butterfly prototype, or an asymmetric butterfly placebo prototype that lacked the springs in the active prototype. Investigators and subjects were blinded as to whether the butterfly-shaped strips were the active or placebo strips. The currently marketed BRNS clear strip was compared with the butterfly placebo because subject awareness of the shape of the strip was not expected to influence efficacy while the subject was asleep. Each strip was applied to the outside of the nose, across the bridge from alar crease to alar crease, according to dispensing instructions that were provided. Subjects used their assigned strip at home every night, for approximately 8 h but no more than 12 h per night, for 2 weeks.

The GSK Consumer Healthcare Biostatistics Department generated the randomization schedule. All subjects were assigned numbers at randomization in consecutive ascending numerical order at one site and consecutive descending numerical order at the second site.

Subjects scored their perceptions of nasal breathing and congestion in a daily diary at home, while lying in a supine position, both before and after applying the strip at bedtime and before and after removing the strip upon waking. Nasal breathing was scored using a 100-mm visual analog scale (VAS) ranging from 0 = extremely difficult to breathe through my nose to 100 = extremely easy to breathe through my nose. Nasal stuffiness was rated on a categorical scale of 0 = no symptoms, 1 = mild symptoms (symptoms clearly present, but minimal awareness and easily tolerated), 2 = moderate symptoms (definite awareness of symptoms that are bothersome but tolerable), and 3 = severe symptoms (symptoms that are hard to tolerate, cause interference with activities or daily living) and on a 100-mm VAS ranging from 0 = nose is extremely blocked to 100 = nose is extremely clear. After applying the nasal strip, subjects rated how breathing felt on an 11-point categorical scale where − 5 = much worse, 0 = same, and 5 = much better; they used the same scale to rate how breathing felt after strip removal.

Subjects visited the study site at baseline, day 7, and day 14 to complete three validated questionnaires: the Pittsburgh Insomnia Rating Scale (PIRS) [18, 19], the Nocturnal Rhinoconjunctivitis Quality of Life Questionnaire (NRQLQ) [20], and the Congestion Quantifier Seven-Item Test (CQ7) [21].

### Ethical considerations

This study was reviewed and approved by an Institutional Review Board (Allendale Investigational Review Board, Old Lyme, CT, USA) and conducted in accordance with Declaration of Helsinki requirements. All subjects provided written informed consent at screening.

### Study population

Subjects were recruited from the general population via advertising or referral. To participate, they had to be ≥ 18 years of age and in good general health, have leptorrhine noses with nocturnal nasal congestion on all or nearly all nights for at least the last year, and report trouble sleeping. A leptorrhine nose was defined as ≥ 45 on the nasal tip protrusion index, which is the ratio of the protrusion of the nose from the face relative to the width of the nose (measured as the length of the columella divided by the width of the alar cartilage) × 100. This inclusion criterion was used because nasal resistance is likely to be greater in leptorrhine noses [22].

Exclusion criteria included allergy or intolerance to the study materials or adhesive bandages, sleep apnea or other major sleep disorder, upper respiratory tract infection, severe deviated septum, nasal polyps, structural abnormality, and treatment for sleep-disordered breathing. Potential subjects with skin conditions that could preclude use of the device (e.g., skin cancer, chronic skin condition, eczema, open sores, sunburn, or irritation on face/nose) were excluded, as were those with a nontypical sleep schedule (e.g., shift work) or plans to travel across time zones during the study period. Current use of prescription or nonprescription medications that affect sleep or nasal congestion was prohibited, as was use of any intranasally administered medications. Those with current alcohol abuse (regular consumption of > 3 drinks/day), recent history (within last 2 years) of substance or alcohol abuse, positive urine drug screen for drugs of abuse, or regular consumption of > 5 cups per day of caffeinated beverages were excluded. Additional exclusion criteria included severe, unstable disease states, pain syndromes, or any medical/surgical condition that would increase the risk of harm to study participants or interfere with data interpretation. Female subjects of childbearing potential had to be practicing a reliable method of contraception, and must not be pregnant or lactating. Individuals who had participated in another study or received an investigational drug within 30 days of the qualification phase, and employees of the sponsor or study site and their immediate family members, were not eligible to participate.

Those who met the eligibility criteria and provided informed consent had to complete a 1-week, at-home, baseline qualification phase. Subjects who scored nasal openness ≤ 70 on a 100-point VAS, where 0 = extremely blocked and 100 = extremely open, on ≥ 4 of 7 nights before bedtime qualified for randomization into the 2-week in-home treatment phase of the study. All corticosteroids (irrespective of route of administration); intranasal cromolyn; intranasal, oral, and ocular antihistamines; other nasal dilators; topical and oral decongestants; intranasal, oral, and inhaled anticholinergics; long-acting beta agonists; and oral antileukotrienes were discontinued and washed out prior to the qualification phase. Subjects were instructed to abstain from consuming alcohol within 8 h of bedtime, maintain consistent sleep and exercise routines, and discontinue use of any lubricating sprays/rinses and throat strips before the baseline qualification phase.

### Efficacy outcomes

Efficacy was evaluated based on changes from baseline for each question and domain on the PIRS, NRQLQ, and CQ7 at weeks 1 and 2, as well as the number and proportion of subjects showing improvement. Efficacy was also evaluated based on changes in VAS ratings of nasal breathing and congestion over weeks 1 and 2. The relationship between the change in these VAS ratings and the change in PIRS, NRQLQ, and CQ7 ratings was explored.

### Safety

Adverse events (AEs) and serious AEs were monitored throughout the study. AEs were graded for severity, and relationship to treatment was assessed by the investigator. Incidents consisting of device malfunction or deterioration or inadequacy of the labeling/instructions for use that could potentially lead to the death or serious deterioration in health of the user or other persons were also monitored.

### Statistical analyses

No power analysis was performed for this exploratory study. We anticipated that screening 90 subjects would allow for enrollment and completion of the study by 60 subjects (20 in each treatment group), with few dropouts.

There were two analysis populations. The safety population consisted of all subjects who were randomized and received any treatment. The intent-to-treat (ITT) population included all randomized subjects who had at least one post-baseline efficacy assessment.

Composite variables were derived as the sum of the item scores within each composite variable. Analysis of covariance (ANCOVA) was used to compare changes from baseline to days 7 and 14, with baseline and site as covariates.

Statistical comparisons were made for four questions from PIRS pertaining to waking after sleep onset, unrefreshing sleep, sleep quality, and satisfaction with sleep; four composite variables on NRQLQ (sleep problems, sleep time problems, symptoms on waking in the morning, and practical problems); and a composite of all seven questions of CQ7. Pairwise multiple comparisons were used to compare the three treatments. Within-treatment improvement from baseline was tested using least square (LS) means compared with zero from the same ANCOVA model described above. All tests were performed at the 5% significance level, and 95% confidence intervals (CIs) were determined.

An additional ad hoc analysis was conducted to analyze three composite variables on the PIRS, explore responses of patients at risk for sleep apnea (defined as meeting at least two of the following three criteria on the Berlin Questionnaire: score ≥ 2 on sum of questions 1–5, score ≥ 2 on sum of questions 6–9; and response of 1 on question 10 or body mass index > 30 kg/m^2^), and determine the incidence of subjects showing any improvement after treatment in their subjective perception of response on the PIRS, NRQLQ, and perception of nasal patency from the daily diary questions. The three composite variables on the PIRS consisted of daytime distress (questions 1‒12), nighttime sleep parameters (questions 13‒16), and quality of life (questions 17‒20). Number and percentage of subjects showing improvement after 7 and 14 days were calculated for sleep quality compared with most people (PIRS question 17), satisfaction with sleep (PIRS question 18), PIRS nighttime sleep parameters and quality-of-life domains, and NRQLQ sleep problems and symptoms on waking domains. Number and percentage of subjects showing any improvement in daily diary ratings on the first night were also calculated. Changes from baseline to days 7 and 14 were analyzed using ANCOVA with treatment as a fixed effect and site and baseline as covariates. Other statistical analyses used in the ad hoc analysis were the same as described for the primary analysis above.

The same ANCOVA model from the primary analysis was also used to compare changes in the degree of perception of nasal breathing and nasal congestion on the daily diary ratings on days 1, 3, 7, and 14. For the two VAS ratings and the 4-point categorical rating of nasal stuffiness, ratings after the strip was applied were compared with ratings before strip application at bedtime, and ratings while wearing the strip were compared with ratings after strip removal upon awakening. Since the 11-point categorical rating of how breathing felt after the strip was applied was performed only during treatment, there was no comparison with before or after use for this outcome.

No imputations were made for missing data or dropouts.

## Results

### Study population

**Fig. 1:**
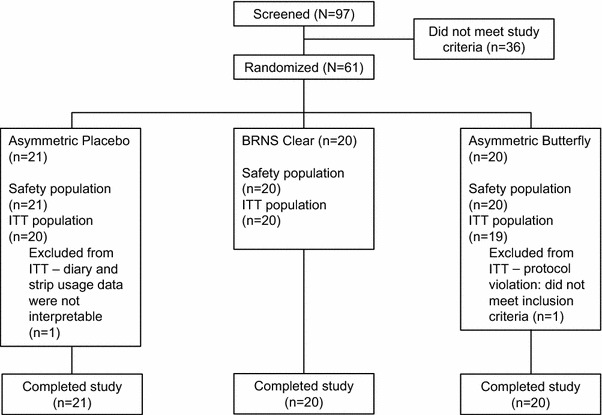
Subject disposition. *BRNS* Breathe Right Nasal Strip, *ITT* intent-to-treat

A total of 97 individuals were screened, and 61 subjects were randomized, all of whom completed the study (Fig. 1). There were 59 subjects included in the ITT population. Of the two excluded subjects, one was randomized despite not meeting eligibility criteria and one had unreliable and uninterpretable diary and strip usage records.

Mean age of the study population was 44.4 years (range 18–73 years); all subjects were white and most were female (50/61, 82.0%) (Table 1). Many subjects had more than one cause of nasal congestion; it was attributed to rhinitis/upper respiratory tract infection in 57 subjects (93.4%), structural abnormality in 23 (37.7%), and other in four cases (6.6%). The mean [standard deviation (SD)] nasal openness score was 28.1 (14.2).

**Table 1:** Baseline demographics

Parameter	Asymmetric placebo (n = 21)	BRNS clear (n = 20)	Asymmetric butterfly (n = 20)
Age, years			
Mean (SD)	44.5 (9.6)	46.1 (10.2)	42.5 (14.0)
Median (range)	45.0 (27‒63)	44.0 (33‒73)	45.0 (18‒71)
Sex, n (%)			
Male	4 (19.0)	2 (10.0)	5 (25.0)
Female	17 (81.0)	18 (90.0)	15 (75.0)
Race, n (%)			
White	21 (100.0)	20 (100.0)	20 (100.0)

*BRNS* Breathe Right Nasal Strip, *SD* standard deviation

### Efficacy results

#### Questionnaires

All three treatment groups had improved PIRS outcomes at most time points compared with baseline. The asymmetric butterfly prototype showed significant (*P *< 0.05) improvement compared with placebo on satisfaction with sleep at 1 and 2 weeks of use and on sleep quality at 2 weeks (Table 2). The BRNS clear strip showed significant (*P *< 0.05) improvement versus placebo on satisfaction with sleep at 1 week of use. The asymmetric butterfly and the BRNS clear strips produced comparable results on all PIRS outcomes.

**Table 2:** Pittsburgh Insomnia Rating Scale results (ITT population)

PIRS item	Asymmetric placebo (n = 20)	BRNS clear (n = 20)	Asymmetric butterfly (n = 19)
***≥ 1 Awakenings after getting to sleep*** ^a^			
Mean (SD) at baseline	2.45 (0.61)	2.10 (0.72)	2.16 (0.83)
*Day 7*			
LS mean change from baseline (95% CI)	− 0.69 (− 0.99 to − 0.40)	− 0.91 (− 1.21 to − 0.61)	− 1.03 (− 1.34 to − 0.72)
*P* value for comparison with placebo	–	*P *= 0.2879	*P *= 0.1029
*P* value for comparison with BRNS	–	–	*P *= 0.5456
*Day 14*			
LS mean change from baseline (95% CI)	− 0.84 (− 1.14 to − 0.54)	− 1.05 (− 1.36 to − 0.74)	− 1.16 (− 1.48 to − 0.84)
*P* value for comparison with placebo	–	*P *= 0.3348	*P *= 0.1429
*P* value for comparison with BRNS	–	–	*P *= 0.5958
***Sleep that is not fully refreshing*** ^a^			
Mean (SD) at baseline	2.25 (0.91)	2.30 (0.80)	2.37 (0.60)
*Day 7*			
LS mean change from baseline (95% CI)	− 0.71 (− 1.07 to − 0.34)	− 1.00 (− 1.37 to − 0.63)	− 0.95 (− 1.33 to − 0.56)
*P* value for comparison with placebo	–	*P *= 0.2364	*P *= 0.3457
*P* value for comparison with BRNS	–	–	*P *= 0.8213
*Day 14*			
LS mean change from baseline (95% CI)	− 0.92 (− 1.30 to − 0.54)	− 1.31 (− 1.69 to − 0.92)	− 1.10 (− 1.50 to − 0.70)
*P* value for comparison with placebo	–	*P *= 0.1375	*P *= 0.4795
*P* value for comparison with BRNS	–	–	*P *= 0.4425
***Sleep quality compared to most people*** ^b^			
Mean (SD) at baseline	2.25 (0.72)	2.30 (0.66)	2.32 (0.48)
*Day 7*			
LS mean change from baseline (95% CI)	− 0.35 (− 0.63 to − 0.07)	− 0.72 (− 1.01 to − 0.43)	− 0.54 (− 0.84 to − 0.24)
*P* value for comparison with placebo	–	*P *= 0.0591	*P *= 0.3369
*P* value for comparison with BRNS	–	–	*P *= 0.3553
*Day 14*			
LS mean change from baseline (95% CI)	− 0.36 (− 0.68 to − 0.04)	− 0.71 (− 1.04 to − 0.39)	− 0.84 (− 1.17 to − 0.50)
*P* value for comparison with placebo	–	*P *= 0.1061	*P *= 0.0337^c^
*P* value for comparison with BRNS	–	–	*P *= 0.5750
***Satisfaction with sleep*** ^b^			
Mean (SD) at baseline	2.30 (0.80)	2.35 (0.67)	2.63 (0.60)
*Day 7*			
LS mean change from baseline (95% CI)	− 0.23 (− 0.50 to 0.03)	− 0.64 (− 0.91 to − 0.38)	− 0.70 (− 0.98 to − 0.42)
*P* value for comparison with placebo	–	*P* = 0.0256^c^	*P *= 0.0151^c^
*P* value for comparison with BRNS	–	–	*P *= 0.7686
*Day 14*			
LS mean change from baseline (95% CI)	− 0.46 (− 0.76 to − 0.16)	− 0.87 (− 1.18 to − 0.57)	− 0.95 (− 1.27 to − 0.63)
*P* value for comparison with placebo	–	*P* = 0.0503	*P *= 0.0250^c^
*P* value for comparison with BRNS	–	–	*P *= 0.7092

*BRNS* Breathe Right Nasal Strip, *CI* confidence interval, *ITT* intent-to-treat, *LS* least square, *PIRS* Pittsburgh Insomnia Rating Scale, *SD* standard deviation

^a^ Scored on a 4-point scale where 0 = not at all bothered, 1 = slightly bothered, 2 = moderately bothered, and 3 = severely bothered

^b^ Scored on a 4-point scale where 0 = excellent, 1 = good, 2 = fair, and 3 = poor

^c^ Significant difference between treatments

On the ad hoc analysis, in the overall ITT population, the butterfly prototype and BRNS clear strips produced significantly (*P *< 0.05) greater improvement from baseline to day 7 than the placebo strip for PIRS nighttime sleep parameters (LS mean changes − 2.03, − 2.11, and − 1.05, respectively) and quality of life (− 2.68, − 3.12, and − 1.26, respectively). At day 14, the asymmetric butterfly prototype showed significantly greater improvement over baseline compared with the placebo strip only for quality of life (− 3.85 vs − 1.74; *P *= 0.0046).

In the subset of subjects at risk for sleep apnea, both active strips produced significantly (*P *< 0.05) greater improvements from baseline versus placebo on all three composite PIRS variables on day 7. At day 14, both active strips showed improvements in quality of life versus the placebo strip in the subgroup at risk for sleep apnea, and the butterfly prototype was also significantly better than placebo with regard to daytime distress (see Additional file 1: Table S1).

All three treatment groups had improved NRQLQ outcomes compared with baseline. Compared with placebo, both the asymmetric butterfly prototype (*P *= 0.0099) and the BRNS clear strip (*P *= 0.0298) more effectively improved symptoms on waking in the morning at 1 week of use (Table 3). The BRNS clear strip was more effective than placebo (*P *= 0.0221) in improving sleep problems at 1 week of use. The asymmetric butterfly and the BRNS clear strips produced comparable results on all NRQLQ outcomes.

**Table 3:** Nocturnal Rhinoconjunctivitis Quality of Life Questionnaire results (ITT population)

NRQLQ item^a^	Asymmetric placebo (n = 20)	BRNS clear (n = 20)	Asymmetric butterfly (n = 19)
***Sleep problems*** ^b^			
Mean (SD) at baseline	15.10 (6.66)	14.30 (4.57)	13.63 (5.96)
*Day 7*			
LS mean change from baseline (95% CI)	− 4.19 (− 6.18 to − 2.21)	− 7.40 (− 9.44 to − 5.36)	− 6.56 (− 8.71 to − 4.42)
*P* value for comparison with placebo	–	*P *= 0.0221^c^	*P *= 0.0942
*P* value for comparison with BRNS	–	–	*P *= 0.5462
*Day 14*			
LS mean change from baseline (95% CI)	− 5.74 (− 7.74 to − 3.74)	− 7.62 (− 9.68 to − 5.57)	− 7.05 (− 9.21 to − 4.89)
*P* value for comparison with placebo		*P *= 0.1739	*P *= 0.3524
*P* value for comparison with BRNS			*P *= 0.6804
***Sleep time problems*** ^d^			
Mean (SD) at baseline	15.75 (6.99)	15.45 (5.99)	15.84 (7.95)
*Day 7*			
LS mean change from baseline (95% CI)	− 5.64 (− 8.09 to − 3.19)	− 7.49 (− 9.99 to − 5.00)	− 8.21 (− 10.81 to − 5.61)
*P* value for comparison with placebo	–	*P *= 0.2722	*P *= 0.1354
*P* value for comparison with BRNS	–	–	*P *= 0.6731
*Day 14*			
LS mean change from baseline (95% CI)	− 7.05 (− 9.49 to − 4.61)	− 8.75 (− 11.24 to − 6.26)	− 6.77 (− 9.36 to − 4.18)
*P* value for comparison with placebo	–	*P *= 0.3136	*P *= 0.8677
*P* value for comparison with BRNS	–	–	*P *= 0.2463
***Symptoms on waking in the morning*** ^e^			
Mean (SD) at baseline	16.55 (6.25)	15.80 (4.42)	14.74 (4.94)
*Day 7*			
LS mean change from baseline (95% CI)	− 4.07 (− 6.13 to − 2.02)	− 7.20 (− 9.28 to − 5.11)	− 7.91 (− 10.09 to − 5.72)
*P* value for comparison with placebo	–	*P *= 0.0298^c^	*P *= 0.0099^c^
*P* value for comparison with BRNS	–	–	*P *= 0.6195
*Day 14*			
LS mean change from baseline (95% CI)	− 5.32 (− 7.33 to − 3.30)	− 7.86 (− 9.90 to − 5.81)	− 8.00 (− 10.14 to − 5.86)
*P* value for comparison with placebo	–	*P *= 0.0695	*P *= 0.0614
*P* value for comparison with BRNS	–	–	*P *= 0.9183
***Practical problems*** ^f^			
Mean (SD) at baseline	7.25 (4.99)	6.05 (4.36)	6.53 (5.21)
*Day 7*			
LS mean change from baseline (95% CI)	− 2.28 (− 3.51 to − 1.04)	− 2.77 (− 4.02 to − 1.52)	− 3.08 (− 4.38 to − 1.78)
*P* value for comparison with placebo	–	*P *= 0.5564	*P *= 0.3500
*P* value for comparison with BRNS	–	–	*P *= 0.7211
*Day 14*			
LS mean change from baseline (95% CI)	− 3.21 (− 4.34 to − 2.07)	− 3.59 (− 4.74 to − 2.44)	− 3.05 (− 4.25 to − 1.86)
*P* value for comparison with placebo	–	*P *= 0.6223	*P *= 0.8454
*P* value for comparison with BRNS	–	–	*P *= 0.4947

*BRNS* Breathe Right Nasal Strip, *CI* confidence interval, *ITT* intent-to-treat, *LS* least square, *NRQLQ* Nocturnal Rhinoconjunctivitis Quality of Life Questionnaire, *SD* standard deviation

^a^ Items in individual domains were scored on a 7-point scale where 0 = not troubled, 1 = hardly troubled, 2 = somewhat troubled, 3 = moderately troubled, 4 = quite a bit troubled, 5 = very troubled, and 6 = extremely troubled

^b^ Computed as the sum of sleep problems domain items 1, 2, 3, and 4

^c^ Significant difference between treatments

^d^ Computed as the sum of sleep time problems domain items 5, 6, 7, 8, and 9

^e^ Computed as the sum of waking in the morning domain items 10, 11, 12, and 13

^f^ Computed as the sum of practical problems domain items 14, 15, and 16

In the subgroup of subjects at risk for sleep apnea, the asymmetric butterfly prototype and BRNS clear strip resulted in significantly greater improvement from baseline to day 7 compared with placebo for sleep problems (LS mean change − 8.01, − 7.24, and − 2.20, respectively; *P *< 0.01 for both comparisons) and symptoms on waking in the morning (− 8.88, − 7.70, and − 3.09, respectively; *P * = 0.0043 for butterfly prototype and *P* = 0.0165 for BRNS clear vs placebo). Only the butterfly prototype was significantly better than placebo for change from baseline to day 7 for sleep time problems (− 9.29 vs − 4.67; *P * = 0.0426) and change from baseline to day 14 for symptoms on waking in the morning (− 9.45 vs − 4.76; *P *= 0.0218).

All three treatment groups showed improvements in CQ7 compared with baseline. There were no between-treatment differences in subjective response to nasal dilation on the CQ7 (Table 4).

**Table 4:** Congestion Quantifier 7 results (ITT population)

CQ7^a^	Asymmetric placebo (n = 20)	BRNS clear (n = 20)	Asymmetric butterfly (n = 19)
Mean (SD) at baseline	19.20 (5.05)	18.70 (4.35)	16.79 (5.31)
*Day 7*			
LS mean change from baseline (95% CI)	− 5.14 (− 7.53 to − 2.74)	− 6.85 (− 9.28 to − 4.41)	− 8.18 (− 10.75 to − 5.62)
*P* value for comparison with placebo	–	*P* = 0.2986	*P* = 0.0764
*P* value for comparison with BRNS	–	–	*P* = 0.4258
*Day 14*			
LS mean change from baseline (95% CI)	− 5.10 (− 7.37 to − 2.83)	− 8.04 (− 10.34 to − 5.73)	− 6.92 (− 9.35 to − 4.49)
*P* value for comparison with placebo	–	*P* = 0.0623	*P* = 0.2607
*P* value for comparison with BRNS	–	–	*P* = 0.4820

*BRNS* Breathe Right Nasal Strip, *CI* confidence interval, *CQ7* Congestion Quantifier 7, *ITT* intent-to-treat, *LS* least square, *SD* standard deviation

^a^ CQ7 was calculated by adding the responses to the seven items on the CQ7 questionnaire for each subject. Each question was rated on a scale of 0 = none of the time, 1 = a little of the time, 2 = some of the time, 3 = most of the time, and 4 = all of the time

#### Results from daily diary ratings at bedtime

The asymmetric butterfly strip significantly (*P *= 0.0417) improved nasal stuffiness compared with placebo on the VAS rating at day 14 only and was comparable to the BRNS clear strip for this outcome (Table 5). The asymmetric butterfly did not significantly reduce ratings of nasal stuffiness compared with placebo or the BRNS clear strip on the 4-point categorical rating (Table 5). The BRNS clear strip significantly (*P *< 0.05) reduced nasal stuffiness compared with placebo on a majority of the categorical and VAS ratings (Table 5).

**Table 5:** Subject diary ratings of nasal stuffiness before and after strip application at bedtime (ITT population)

	Categorical ratings^a^			VAS ratings^b^		
	Asymmetric placebo (n = 20)	BRNS clear (n = 20)	Asymmetric butterfly (n = 19)	Asymmetric placebo (n = 20)	BRNS clear (n = 20)	Asymmetric butterfly (n = 19)
*Day 1*						
Mean (SD) before strip application	2.00 (0.56)	1.95 (0.39)	1.79 (0.54)	37.74 (17.14)	27.90 (20.46)	35.89 (14.64)
Mean (SD) after strip application	1.80 (0.70)	1.10 (0.79)	1.26 (0.56)	45.84 (23.06)	61.45 (25.85)	57.68 (22.58)
LS mean change after vs before strip application (95% CI); *P* value	− 0.11 (− 0.40 to 0.18); *P* = 0.4637	− 0.77 (− 1.06 to − 0.47); *P* < 0.0001	− 0.50 (− 0.81 to − 0.19); *P* = 0.0019	7.66 (− 2.93 to 18.25); *P* = 0.1528	29.10 (18.73 to 39.47); *P* < 0.0001	18.80 (7.86 to 29.74); *P* = 0.0011
*P* value for comparison with placebo	–	*P* = 0.0016^c^	*P* = 0.0580	–	*P* = 0.0040^c^	*P* = 0.1236
*P* value for comparison with BRNS	–	–	*P* = 0.1986	–	–	*P* = 0.1561
*Day 3*						
Mean (SD) before strip application	1.90 (0.55)	1.90 (0.64)	1.58 (0.69)	38.32 (19.29)	30.05 (20.41)	46.42 (24.13)
Mean (SD) after strip application	1.60 (0.75)	1.16 (0.90)	1.16 (0.69)	49.74 (22.10)	59.25 (27.47)	62.16 (24.85)
LS mean change after vs before strip application (95% CI); *P* value	− 0.20 (− 0.51 to 0.11); *P* = 0.2047	− 0.66 (− 0.99 to − 0.33); *P* = 0.0002	− 0.41 (− 0.75 to − 0.07); *P* = 0.0176	9.40 (− 1.23 to 20.04); *P* = 0.0819	23.50 (12.86 to 34.13); *P* < 0.0001	16.75 (5.57 to 27.93); *P* = 0.0040
*P* value for comparison with placebo	–	*P* = 0.0377^c^	*P* = 0.3447	–	*P* = 0.0539	*P* = 0.3152
*P* value for comparison with BRNS	–	–	*P* = 0.2733	–	–	*P* = 0.3678
*Day 7*						
Mean (SD) before strip application	1.70 (0.73)	1.75 (0.55)	1.63 (0.60)	37.74 (22.42)	30.75 (21.75)	42.05 (21.37)
Mean (SD) after strip application	1.55 (0.89)	1.00 (0.80)	1.16 (0.69)	48.63 (26.87)	60.85 (27.31)	64.95 (21.71)
LS mean change after vs before strip application (95% CI); *P* value	− 0.12 (− 0.38 to 0.14); *P *= 0.3512	− 0.71 (− 0.97 to − 0.45); *P* < 0.0001	− 0.44 (− 0.71 to − 0.16); *P* = 0.0022	8.62 (− 1.40 to 18.64); *P* = 0.0903	25.43 (15.50 to 35.35); *P* < 0.0001	21.43 (11.08 to 31.79); *P* = 0.0001
*P* value for comparison with placebo	–	*P* = 0.0015^c^	*P* = 0.0815	–	*P* = 0.0153^c^	*P* = 0.0635
*P* value for comparison with BRNS	–	–	*P* = 0.1345	–	–	*P* = 0.5600
*Day 14*						
Mean (SD) before strip application	1.58 (0.61)	1.40 (0.60)	1.61 (0.70)	43.84 (22.32)	40.90 (24.73)	45.78 (23.99)
Mean (SD) after strip application	1.35 (0.79)	0.85 (0.88)	1.06 (0.88)	53.21 (25.94)	68 (25.51)	69.56 (24.12)
LS mean change after vs before strip application (95% CI); *P* value	− 0.09 (− 0.45 to 0.27); *P* = 0.6129	− 0.50 (− 0.83 to − 0.17); *P* = 0.0036	− 0.45 (− 0.80 to − 0.09); *P* = 0.0142	5.44 (− 4.86 to 15.74); *P* = 0.2942	21.47 (11.33 to 31.60); *P* < 0.0001	20.10 (9.36 to 30.85); *P* = 0.0004
*P* value for comparison with placebo	–	*P* = 0.0786	*P* = 0.1356	–	*P* = 0.0230^c^	*P* = 0.0417^c^
*P* value for comparison with BRNS	–	–	*P* = 0.8063	–	–	*P* = 0.8451

*BRNS* Breathe Right Nasal Strip, *CI* confidence interval, *ITT* intent-to-treat, *LS* least square, *SD* standard deviation, *VAS* visual analog scale

^a^ Subjects rated how stuffed their noses felt on a scale of 0 = no symptoms, 1 = mild symptoms, 2 = moderate symptoms, and 3 = severe symptoms

^b^ VAS scale of 0 = nose is extremely blocked to 100 = nose is extremely clear

^c^ Significant difference between treatments

The asymmetric butterfly strip significantly improved ease of breathing compared with placebo on day 14 on both the categorical (*P *= 0.0438) and VAS (*P *= 0.0385) ratings (Table 6). The BRNS clear strip significantly (*P *< 0.05) improved breathing compared with placebo at days 1, 3, 7, and 14 on both categorical and VAS ratings, and significantly (*P *= 0.0292) improved breathing compared with the asymmetric butterfly at day 7 on the categorical rating (Table 6).

**Table 6:** Subject diary ratings of breathing before and after strip application at bedtime (ITT population)

	Categorical ratings^a^				VAS ratings^b^		
	Asymmetric placebo (n = 20)	BRNS clear (n = 20)	Asymmetric butterfly (n = 19)		Asymmetric placebo (n = 20)	BRNS clear (n = 20)	Asymmetric butterfly (n = 19)
*Day 1*				*Day 1*			
Mean (SD) before strip application	NA	NA	NA	Mean (SD) before strip application	34.58 (15.80)	27.85 (20.58)	34.00 (14.82)
Mean (SD) after strip application	1.20 (1.47)	2.45 (1.40)	1.42 (2.12)	Mean (SD) after strip application	43.11 (24.07)	61.55 (26.72)	55.00 (24.87)
LS mean after strip application	1.07	2.29	1.24	LS mean change after vs before strip application (95% CI); *P* value	7.37 (− 3.73 to 18.46); *P* = 0.1886	30.24 (19.38 to 41.10); *P* < 0.0001	19.21 (7.89 to 30.54); *P* = 0.0013
*P* value for comparison with placebo	–	*P* = 0.0254^c^	*P* = 0.7624	*P* value for comparison with placebo	–	*P* = 0.0032^c^	*P* = 0.1157
*P* value for comparison with BRNS	–	–	*P* = 0.0546	*P* value for comparison with BRNS	–	–	*P* = 0.1420
*Day 3*				*Day 3*			
Mean (SD) before strip application	NA	NA	NA	Mean (SD) before strip application	37.95 (19.84)	29.35 (20.30)	45.11 (23.87)
Mean (SD) after strip application	1.20 (1.36)	2.40 (1.67)	1.74 (1.73)	Mean (SD) after strip application	48.53 (20.67)	58.90 (26.64)	61.42 (24.74)
LS mean after strip application	0.96	2.10	1.39	LS mean change after vs before strip application (95% CI); *P* value	8.67 (− 1.65 to 18.99); *P* = 0.0978	23.70 (13.42 to 33.97); *P* < 0.0001	17.10 (6.26 to 27.95); *P* = 0.0026
*P* value for comparison with placebo	–	*P* = 0.0208^c^	*P* = 0.3799	*P* value for comparison with placebo	–	*P* = 0.0348^c^	*P* = 0.2341
*P* value for comparison with BRNS	–	–	*P* = 0.1494	*P* value for comparison with BRNS	–	–	*P* = 0.3625
*Day 7*				*Day 7*			
Mean (SD) before strip application	NA	NA	NA	Mean (SD) before strip application	36.37 (21.95)	32.10 (22.39)	40.53 (18.23)
Mean (SD) after strip application	1.20 (1.47)	2.80 (1.40)	1.79 (1.48)	Mean (SD) after strip application	47.42 (26.74)	58.95 (28.54)	61.11 (21.79)
LS mean after strip application	1.09	2.66	1.62	LS mean change after vs before strip application (95% CI); *P* value	8.75 (− 1.43 to 18.92); *P* = 0.0905	23.25 (13.21 to 33.29); *P* < 0.0001	18.89 (8.40 to 29.38); *P* = 0.0007
*P* value for comparison with placebo	–	*P* = 0.0011^c^	*P* = 0.2497	*P* value for comparison with placebo	–	*P* = 0.0370^c^	*P* = 0.1458
*P* value for comparison with BRNS	–	–	*P* = 0.0292^c^	*P* value for comparison with BRNS	–	–	*P* = 0.5278
*Day 14*				*Day 14*			
Mean (SD) before strip application	NA	NA	NA	Mean (SD) before strip application	43.21 (24.00)	39.90 (24.07)	45.89 (22.29)
Mean (SD) after strip application	0.89 (1.41)	2.70 (1.66)	2.00 (1.72)	Mean (SD) after strip application	51.05 (25.39)	68.70 (26.64)	67.56 (23.61)
LS mean after strip application	0.74	2.54	1.82	LS mean change after vs before strip application (95% CI); *P* value	4.15 (− 5.70 to 14.01); *P* = 0.4017	23.52 (13.82 to 33.23); *P* < 0.0001	18.43 (8.14 to 28.72); *P* = 0.0007
*P* value for comparison with placebo	–	*P* = 0.0009^c^	*P* = 0.0438^c^	*P* value for comparison with placebo	–	*P* = 0.0046^c^	*P* = 0.0385^c^
*P* value for comparison with BRNS	–	–	*P* = 0.1693	*P* value for comparison with BRNS	–	–	*P* = 0.4483

*BRNS* Breathe Right Nasal Strip, *CI* confidence interval, *ITT* intent-to-treat, *LS* least square, *NA* not applicable; *SD* standard deviation, *VAS* visual analog scale

^a^ Subjects rated how breathing felt after they applied the strip using a scale of − 5 = much worse, 0 = same, and 5 = much better

^b^ VAS scale of 0 = extremely difficult to breathe to 100 = extremely easy to breathe

^c^ Significant difference between treatments

In the ad hoc analysis, after strip application, more subjects using either active strip experienced some improvement on the first night compared with placebo (see Additional file 2: Table S2).

#### Results from daily diary ratings upon awakening

As expected, comparisons of categorical ratings of symptom severity before and after strip removal showed that the BRNS clear group had a significant return of symptoms after removing the strip in the morning at days 3, 7, and 14, although the changes were not significantly different across the treatment groups. Similarly, the before and after strip removal comparisons of VAS ratings showed that the BRNS clear group experienced a significant return of nasal stuffiness after the strip was removed at days 3, 7, and 14, which was significantly different compared with both placebo and the asymmetric butterfly at days 3 and 7. These were the only between-treatment differences on this outcome. The VAS and categorical ratings of nasal stuffiness for the butterfly strip arm did not differ significantly after removal of the strip in the morning (see Additional file 3: Table S3).

After removing the strip upon awakening in the morning, breathing significantly worsened to a greater extent in the BRNS clear group than in the asymmetric butterfly group at days 7 (*P *= 0.0381) and 14 (*P *= 0.0434) based on the categorical ratings (see Additional file 4: Table S4). Similarly, the BRNS clear group experienced a significant decrease in VAS ratings for ease of breathing after removal of the strip (*P *< 0.01) at all four time points evaluated. Ease of breathing was not significantly changed after strip removal at any time in the asymmetric butterfly group. At days 3 (*P *= 0.0075) and 7 (*P *= 0.0029), the decrease in ease of breathing ratings was significantly greater after strip removal in the BRNS clear group than in the asymmetric butterfly group.

### Safety results

Overall, the nasal strips were well tolerated. The only AE was a mild nasal scab secondary to asymmetric butterfly placebo strip removal on day 4 of treatment, which was considered treatment related. It resolved on day 15, and the subject completed the study. There were no serious or severe AEs.

## Discussion

In this exploratory study, both the asymmetric butterfly prototype and the currently marketed BRNS clear strip significantly improved some subjective measures of nasal congestion and sleep compared with placebo in subjects with moderate to severe chronic nocturnal nasal congestion and sleep difficulties. The two active strips produced results that were comparable to each other on most outcomes. The nasal strips were well tolerated.

On the PIRS, the improvement in “sleep quality as compared to most people” with the asymmetric butterfly prototype was more than twice that with placebo on day 14 (− 36.5% vs − 15.7%), and improvement in “satisfaction with sleep” was more than twice that of placebo on day 7 (− 26.9% vs − 10.0%) and almost twice that on day 14 (− 36.5% vs − 20.0%). On the NRQLQ, the nasal dilator strips impacted the two domains that measure rhinitis symptoms most likely to be affected by nasal dilation. The BRNS clear strip was superior to placebo on day 7 for the sleep problems domain. Both strips were superior to placebo on the “symptoms on waking in the morning” domain on day 7. The CQ7 did not differentiate between the treatment groups, possibly because it addresses symptoms that nasal dilation may not affect (e.g., sinus pain/pressure, difficulty clearing the nose after repeated blowing). The magnitude of the differences in PIRS and NRQLQ domains between active strips and placebo strips was generally greater in subjects at risk for sleep apnea than in the study population as a whole.

Although there were few statistically significant differences between the BRNS clear strip and the asymmetric butterfly strip on the bedtime diary ratings of nasal stuffiness and breathing, the BRNS clear strip was significantly superior to placebo more frequently than the asymmetric butterfly strip was. Upon removal of the strip, the nose is expected to return to its normal shape; therefore a reduction in nasal patency and return of symptoms (i.e., worsening in the categorical and VAS ratings) after strip removal in the morning was anticipated. As expected, the effects of the BRNS clear strip were temporary and began to reverse after removal. Significant worsening of symptoms after strip removal was observed more frequently with the BRNS clear strip than the butterfly strip, for which the effects were generally unchanged following strip removal. It is possible that some regions of the nose that were pulled by the asymmetric butterfly returned to normal at a slower pace, but this would require further investigation.

While several previous studies have shown improvements in some objective sleep outcomes measured by polysomnography [14, 17, 23], few studies have examined the effects of the BRNS on patient-reported sleep outcomes. The one previous study that reported effects of the BRNS on subjective sleep outcomes used different patient-reported outcomes from those used in the present study. In a 2-week, open-label study of 20 adults aged 22–54 years with at least a 3-month history of snoring, Scharf et al. [13] assessed the effects of the BRNS on sleep using the Stanford Sleepiness Scale (a measure of daytime sleepiness), pre- and post-sleep questionnaires, and a bed-partner post-sleep questionnaire. Subjects received no treatment for the first week and then used the BRNS nightly for the second week. Results showed that the BRNS strip significantly reduced daytime sleepiness on the Stanford Sleepiness Scale; improved self-reported ease of breathing, quality of sleep, sleepiness upon awakening, morning concentration, and number of awakenings; and improved partner reports of snoring loudness and movement. Results from this previous study are generally consistent with our findings that the BRNS improved sleep quality, symptoms upon awakening, and ease of breathing. The fact that effects on those outcomes were shown using different sets of measurement tools lends further support for a genuine therapeutic effect of the nasal dilator strips on these sleep outcomes.

Participants in the current study had nocturnal nasal congestion on all or nearly all nights for at least the last year and related sleep difficulties. As the effects of the nasal dilators are largely limited to the period of use, ongoing, long-term use may be necessary for continued symptom relief. While our study was limited to 2 weeks, a prior randomized, controlled 4-week study of nasal dilator strip use by nonobese patients with mild to moderate sleep-disordered breathing and sleep-maintenance insomnia (N = 91) found that the strip was associated with large improvements in the severity of insomnia and sleep quality, and moderate improvements in sleepiness and quality of life scores on patient-reported outcome scales (Insomnia Severity Index, PSQI, FOSQ, and Quality of Life Enjoyment and Satisfaction Questionnaire) [15]. While longer-term follow-up is lacking, the results of the current trial and the 4-week study show no indication that the potential benefits of nasal dilator strips wane over time with nightly use.

High placebo response was observed in the current study, such that there was a significant improvement over baseline on most outcomes. This may be attributed, in part, to patients liking the experience of applying the strips. It is unknown whether the placebo strip had some active effect despite the absence of the springs. This study lacked a placebo strip matched to the BRNS clear, which was compared, instead, with the asymmetric butterfly-shaped placebo. Subjects in the butterfly placebo group remained unaware of whether they had received a placebo or the active prototype strip. Additional limitations include the large number of study endpoints and the small sample size. This was an exploratory study; therefore, the results are hypothesis generating to enable more targeted research in future well-powered studies.

## Conclusions

This exploratory study suggests that nasal dilator strips potentially improve sleep quality, satisfaction with sleep, sleep problems, symptoms on waking, nasal stuffiness, and ease of breathing. However, the high placebo response precludes drawing definite conclusions. These outcomes should be considered as endpoints for future large placebo-controlled trials. Further research may be needed to identify which patient-reported sleep outcome measures best detect clinically meaningful effects of nasal dilator strips on sleep. In our study, the prototype asymmetric butterfly strip was generally comparable to the currently marketed BRNS clear strip.

## Additional files

**Additional file 1: Table S1.** Composite PIRS measures in subgroup of subjects at risk for sleep apnea (ITT population).

**Additional file 2: Table S2.** Subjects showing any improvement on the daily diary questions after strip application on night 1.

**Additional file 3: Table S3.** Subject diary ratings of nasal stuffiness before and after strip removal upon awakening (ITT population).

**Additional file 4: Table S4.** Subject diary ratings of breathing before and after strip removal upon awakening (ITT population).

## Abbreviations

AE: adverse event
ANCOVA: analysis of covariance
BRNS: Breathe Right Nasal Strip
CI: confidence interval
CQ7: Congestion Quantifier 7
ITT: intent-to-treat
LS: least square
NRQLQ: Nocturnal Rhinoconjunctivitis Quality of Life Questionnaire
PIRS: Pittsburgh Insomnia Rating Scale
SD: standard deviation
VAS: visual analog scale
