# Supplementary material for: Effects of nasal dilator strips on subjective measures of sleep in subjects with chronic nocturnal nasal congestion: a randomized, placebo-controlled trial
Source: Allergy Asthma Clin Immunol. 2018 Aug 27;14:34. doi: 10.1186/s13223-018-0258-5 (PMC6109978; doi:10.1186/s13223-018-0258-5)
Supplement: Supplementary file 1 — Additional file 1: Table S1. Composite PIRS measures in subgroup of subjects at risk for sleep apnea (ITT population). [file 13223_2018_258_MOESM1_ESM.docx]

**Table S1. Composite PIRS measures in subgroup of subjects at risk for sleep apnea (ITT population)**

| **PIRS composite measure** | **Asymmetric placebo (n=12)** | **BRNS clear (n=13)** | **Asymmetric butterfly (n=11)** |
| --- | --- | --- | --- |
| ***Daytime distress^a^*** |  |  |  |
| Mean (SD) at baseline | 18.92 (10.43) | 21.23 (8.08) | 22.64 (10.13) |
| **Day 7** |  |  |  |
| LS mean change from baseline (95% CI) | -3.75 (-7.31 to -0.19) | -9.66 (-12.9 to -6.43) | -11.62 (-15.4 to -7.88) |
| *P* value for comparison with placebo | -- | *P*=0.0151^b^ | *P*=0.0025^b^ |
| *P* value for comparison with BRNS | -- | -- | *P*=0.4135 |
| **Day 14** |  |  |  |
| LS mean change from baseline (95% CI) | -6.64 (-10.4 to -2.86) | -11.47 (-14.9 to -8.05) | -14.07 (-18.0 to -10.1) |
| *P* value for comparison with placebo | -- | *P*=0.0561 | *P*=0.0062^b^ |
| *P* value for comparison with BRNS | -- | -- | *P*=0.3064 |
| ***Nighttime sleep parameters^c^*** |  |  |  |
| Mean (SD) at baseline | 4.92 (3.26) | 4.69 (2.25) | 6.00 (3.58) |
| **Day 7** |  |  |  |
| LS mean change from baseline (95% CI) | -0.70 (-1.67 to 0.27) | -2.62 (-3.51 to -1.72) | -2.48 (-3.50 to -1.46) |
| *P* value for comparison with placebo | -- | *P*=0.0043^b^ | *P*=0.0102^b^ |
| *P* value for comparison with BRNS | -- | -- | *P*=0.8318 |
| **Day 14** |  |  |  |
| LS mean change from baseline (95% CI) | -1.64 (-2.68 to -0.60) | -2.15 (-3.1 to -1.2) | -2.98 (-4.07 to -1.89) |
| *P* value for comparison with placebo | -- | *P*=0.4560 | *P*=0.0645 |
| *P* value for comparison with BRNS | -- | -- | *P*=0.2386 |
| ***Quality of life^d^*** |  |  |  |
| Mean (SD) at baseline | 9.25 (3.05) | 9.92 (1.98) | 10.64 (1.43) |
| **Day 7** |  |  |  |
| LS mean change from baseline (95% CI) | -0.66 (-1.83 to 0.51) | -3.08 (-4.13 to -2.04) | -3.44 (-4.65 to -2.23) |
| *P* value for comparison with placebo | -- | *P*=0.0028^b^ | *P*=0.0013^b^ |
| *P* value for comparison with BRNS | -- | -- | *P*=0.6454 |
| **Day 14** |  |  |  |
| LS mean change from baseline (95% CI) | -1.02 (-2.48 to 0.44) | -3.08 (-4.39 to -1.77) | -4.88 (-6.40 to -3.37) |
| *P* value for comparison with placebo | -- | *P*=0.0347^d^ | *P*=0.0004^d^ |
| *P* value for comparison with BRNS | -- | -- | *P*=0.0683 |

*BRNS* Breathe Right Nasal Strip, *CI* confidence interval, *ITT* intent-to-treat, *LS* least square, *PIRS* Pittsburgh Insomnia Rating Scale

^a^Daytime distress is the sum of items 1 to 12 on PIRS20, each of which is scored on a 4-point scale of 0=not at all bothered, 1=slightly bothered, 2=moderately bothered, 3=severely bothered

^b^Significant difference between treatments

^c^Nighttime sleep parameters is the sum of items 13 to 16 on PIRS20, each of which is scored on a 4-point scale as follows: Q13: 0=less than ½ hour, 1=between ½ hour to 1 hour, 2=between 1 to 3 hours, 3=more than 3 hours or I didn’t sleep. Q14: 0=less than ½ hour or I didn’t wake up, 1=between ½ hour to 1 hour, 2=between 1 to 3 hours, 3=more than 3 hours or I didn’t fall back to sleep. Q15: 0=more than 7 hours, 1=between 4 to 7 hours, 2=between 2 to 4 hours, 3=less than 2 hours or I didn’t sleep. Q16: 0=none or 1 day, 1=on 2 or 3 days, 2=on 4 or 5 days, 3=on 6 or all days

^d^Quality of life is the sum of items 17 to 20 on PIRS20, where each item is scored on a 4-point scale of 0=excellent, 1=good, 2=fair, 3=poor
