# Supplementary material for: Effects of nasal dilator strips on subjective measures of sleep in subjects with chronic nocturnal nasal congestion: a randomized, placebo-controlled trial
Source: Allergy Asthma Clin Immunol. 2018 Aug 27;14:34. doi: 10.1186/s13223-018-0258-5 (PMC6109978; doi:10.1186/s13223-018-0258-5)
Supplement: Supplementary file 2 — Additional file 2: Table S2. Subjects showing any improvement on the daily diary questions after strip application on night 1. [file 13223_2018_258_MOESM2_ESM.docx]

**Table S2. Subjects showing any improvement on the daily diary questions after strip application on night 1**

|  | n/N (%) of subjects | | |
| --- | --- | --- | --- |
| **Daily diary question** | **Asymmetric placebo** | **BRNS clear** | **Asymmetric butterfly** |
| Categorical rating of nasal stuffiness^a^ | 3/20 (15.0) | 13/20 (65.0) | 10/19 (52.6) |
| VAS rating of breathing^b^ | 11/19 (57.9) | 20/20 (100.0) | 15/19 (79.0) |
| VAS rating of nasal congestion^c^ | 13/19 (68.4) | 19/20 (95.0) | 13/18 (72.2) |
| Categorical rating of how breathing felt after applying the strip^d^ | 11/20 (55.0) | 18/20 (90.0) | 12/19 (63.2) |

*BRNS* Breathe Right Nasal Strip, *VAS* visual analog scale

^a^Rated on 4-point scale of 0=no symptoms, 1=mild symptoms, 2=moderate symptoms, 3=severe symptoms

^b^Rated on 100-mm VAS scale from 0=extremely difficult to 100=extremely easy to breathe

^c^Rated on 100-mm VAS scale from 0=nose is extremely blocked to 100=nose is extremely clear

^d^Rated on 11-point scale from -5=much worse, 0=same, and 5=much better
