# Supplementary material for: Effects of nasal dilator strips on subjective measures of sleep in subjects with chronic nocturnal nasal congestion: a randomized, placebo-controlled trial
Source: Allergy Asthma Clin Immunol. 2018 Aug 27;14:34. doi: 10.1186/s13223-018-0258-5 (PMC6109978; doi:10.1186/s13223-018-0258-5)
Supplement: Supplementary file 3 — Additional file 3: Table S3. Subject diary ratings of nasal stuffiness before and after strip removal upon awakening (ITT population). [file 13223_2018_258_MOESM3_ESM.docx]

**Table S3. Subject diary ratings of nasal stuffiness before and after strip removal upon awakening^a^ (ITT population)**

|  | **Categorical ratings^b^** | | | **VAS ratings^c^** | | |
| --- | --- | --- | --- | --- | --- | --- |
|  | **Asymmetric placebo (n=20)** | **BRNS  clear (n=20)** | **Asymmetric butterfly (n=19)** | **Asymmetric placebo (n=20)** | **BRNS  clear (n=20)** | **Asymmetric  butterfly (n=19)** |
| **Day 1** |  |  |  |  |  |  |
| Mean (SD) before strip removal | 1.55 (0.76) | 1.15 (0.75) | 1.22 (0.65) | 45.37 (25.05) | 53.95 (29.88) | 60.22 (21.20) |
| Mean (SD) after strip removal | 1.65 (0.75) | 1.35 (0.67) | 1.33 (0.59) | 42.53 (20.29) | 44.70 (22.18) | 51.56 (22.73) |
| LS mean change after vs before strip removal (95% CI); *P* value | 0.33 (0.04 to 0.61); *P*=0.0247 | 0.19 (-0.09 to 0.47); *P*=0.1701 | 0.16 (-0.13 to 0.45); *P*=0.2815 | -8.75 (-17.3 to  -0.24); *P*=0.0441 | -10.89 (-19.1 to  -2.72); *P*=0.0100 | -7.34 (-16.0 to 1.36); *P*=0.0963 |
| *P* value for comparison with placebo | -- | *P*=0.4869 | *P*=0.3948 | -- | *P*=0.7017 | *P*=0.8100 |
| *P* value for comparison with BRNS | -- | -- | *P*=0.8637 | -- | -- | *P*=0.5304 |
| **Day 3** |  |  |  |  |  |  |
| Mean (SD) before strip removal | 1.45 (0.89) | 0.84 (0.69) | 1.11 (0.74) | 46.89 (23.81) | 61.35 (27.87) | 60.53 (24.69) |
| Mean (SD) after strip removal | 1.55 (0.83) | 1.35 (0.67) | 1.11 (0.58) | 45.47 (22.47) | 44.30 (21.44) | 58.11 (22.65) |
| LS mean change after vs before strip removal (95% CI); *P* value | 0.34 (0.09 to 0.60); *P*=0.0090 | 0.46 (0.20 to 0.72); *P*=0.0009 | 0.11 (-0.15 to 0.37); *P*=0.4020 | -6.52 (-13.8 to 0.81); *P*=0.0800 | -16.80 (-23.7 to  -9.93); *P*<0.0001 | -2.74 (-9.89 to 4.41); *P*=0.4459 |
| *P* value for comparison with placebo | -- | *P*=0.5268 | *P*=0.1831 | -- | *P*=0.0360^d^ | *P*=0.4363 |
| *P* value for comparison with BRNS | -- | -- | *P*=0.0521 | -- | -- | *P*=0.0038^d^ |
| **Day 7** |  |  |  |  |  |  |
| Mean (SD) before strip removal | 1.60 (1.00) | 1.00 (0.65) | 1.00 (0.75) | 45.11 (25.67) | 60.45 (25.60) | 65.05 (20.31) |
| Mean (SD) after strip removal | 1.75 (0.97) | 1.35 (0.67) | 1.11 (0.81) | 45.74 (25.21) | 44.90 (23.86) | 61.95 (21.28) |
| LS mean change after vs before strip removal (95% CI); *P* value | 0.27 (-0.02 to 0.56); *P*=0.0639 | 0.31 (0.03 to 0.586); *P*=0.0282 | 0.067  (-0.22 to 0.35); *P*=0.6394 | -0.96 (-8.42 to 6.50); *P*=0.7981 | -14.03 (-20.9 to  -7.22); *P*=0.0001 | -0.54 (-7.65 to 6.57); *P*=0.8791 |
| *P* value for comparison with placebo | -- | *P*=0.8495 | *P*=0.2964 | -- | *P*=0.0085^d^ | *P*=0.9335 |
| *P* value for comparison with BRNS | -- | -- | *P*=0.1991 | -- | -- | *P*=0.0051^d^ |
| **Day 14** |  |  |  |  |  |  |
| Mean (SD) before strip removal | 1.40 (0.94) | 0.95 (0.83) | 0.89 (0.83) | 44.58 (25.42) | 67.15 (25.27) | 71.39 (21.73) |
| Mean (SD) after strip removal | 1.45 (0.89) | 1.30 (0.66) | 1.17 (0.71) | 44.00 (23.94) | 51.30 (25.28) | 61.00 (22.56) |
| LS mean change after vs before strip removal (95% CI); *P* value | 0.18 (-0.06 to 0.41); *P*=0.1313 | 0.33 (0.10 to 0.56); *P*=0.0055 | 0.24 (0.00 to 0.49); *P*=0.0511 | -5.28  (-14.4 to 3.87); *P*=0.2522 | -14.01 (-22.1 to  -5.90); *P*=0.0011 | -7.31 (-16.0 to 1.36); *P*=0.0965 |
| *P* value for comparison with placebo | -- | *P*=0.3330 | *P*=0.6935 | -- | *P*=0.1447 | *P*=0.7432 |
| *P* value for comparison with BRNS | -- | -- | *P*=0.5741 | -- | -- | *P*=0.2335 |

*BRNS* Breathe Right Nasal Strip, *CI* confidence interval, *ITT* intent-to-treat, *LS* least square, *SD* standard deviation, *VAS* visual analog scale

^a^Upon removal of the strip, the nose is expected to return to its normal shape; therefore, a return of symptoms (ie, worsening in the categorical and VAS ratings) after the device is removed supports a therapeutic effect of the device

^b^Subjects rated how stuffed their noses felt on a scale of 0=no symptoms, 1=mild symptoms, 2=moderate symptoms, and 3=severe symptoms

^c^VAS scale of 0=nose is extremely blocked to 100=nose is extremely clear

^d^Significant difference between treatments
