# Supplementary material for: Effects of nasal dilator strips on subjective measures of sleep in subjects with chronic nocturnal nasal congestion: a randomized, placebo-controlled trial
Source: Allergy Asthma Clin Immunol. 2018 Aug 27;14:34. doi: 10.1186/s13223-018-0258-5 (PMC6109978; doi:10.1186/s13223-018-0258-5)
Supplement: Supplementary file 4 — Additional file 4: Table S4. Subject diary ratings of breathing before and after strip removal upon awakening (ITT population). [file 13223_2018_258_MOESM4_ESM.docx]

**Table S4. Subject diary ratings of breathing before and after strip removal upon awakening^a^ (ITT population)**

|  | **Categorical ratings^b^** | | |  | **VAS ratings^c^** | | |
| --- | --- | --- | --- | --- | --- | --- | --- |
|  | **Asymmetric  placebo (n=20)** | **BRNS  clear (n=20)** | **Asymmetric  butterfly (n=19)** |  | **Asymmetric  placebo (n=20)** | **BRNS  clear (n=20)** | **Asymmetric  butterfly (n=19)** |
| **Day 1** |  |  |  |  |  |  |  |
| Mean (SD) before strip removal | NA | NA | NA | Mean (SD) before strip removal | 45.11 (22.59) | 54.60 (30.41) | 60.44 (20.02) |
| Mean (SD) after strip removal | 0.25 (1.65) | -0.30 (2.06) | 0.50 (2.41) | Mean (SD) after strip removal | 41.47 (20.02) | 44.75 (21.56) | 51.44 (20.57) |
| LS mean after strip removal | 0.11 | -0.48 (-1.45 to 0.48); *P*=0.3221 | 0.30 (-0.72 to 1.32); *P*=0.5601 | LS mean change after vs before strip removal (95% CI);  *P* value | -10.55 (-19.0 to -2.08); *P*=0.0157 | -11.51  (-19.6 to -3.42); *P*=0.0062 | -7.62 (-16.2 to 1.00); *P*=0.0821 |
| *P* value for comparison with placebo | -- | *P*=0.3681 | *P*=0.7717 | *P* value for comparison with placebo | -- | *P*=0.8626 | *P*=0.6143 |
| *P* value for comparison with BRNS | -- | -- | *P*=0.2446 | *P* value for comparison with BRNS | -- | -- | *P*=0.4870 |
| **Day 3** |  |  |  |  |  |  |  |
| Mean (SD) before strip removal | NA | NA | NA | Mean (SD) before strip removal | 48.37 (23.86) | 63.60 (27.58) | 60.21 (24.11) |
| Mean (SD) after strip removal | 0.85 (1.63) | -0.35 (2.18) | 0.68 (1.73) | Mean (SD) after strip removal | 45.53 (23.24) | 44.90 (21.07) | 56.79 (21.58) |
| LS mean after strip removal | 0.61 | -0.65 (-1.50 to 0.21); *P*=0.1364 | 0.34 (-0.55 to 1.23); *P*=0.4424 | LS mean change after vs before strip removal (95% CI);  *P* value | -7.76 (-15.5 to 0.00); *P*=0.0501 | -17.56  (-24.8 to -10.3); *P*<0.0001 | -3.85 (-11.4 to 3.74); *P*=0.3135 |
| *P* value for comparison with placebo | -- | *P*=0.0321^d^ | *P*=0.6426 | *P* value for comparison with placebo | -- | *P*=0.0594 | *P*=0.4439 |
| *P* value for comparison with BRNS | -- | -- | *P*=0.0940 | *P* value for comparison with BRNS | -- | -- | *P*=0.0075^d^ |
| **Day 7** |  |  |  |  |  |  |  |
| Mean (SD) before strip removal | NA | NA | NA | Mean (SD) before strip removal | 46.95 (25.89) | 60.15 (25.52) | 62.63 (19.77) |
| Mean (SD) after strip removal | 0.60 (1.76) | -0.20 (2.12) | 1.16 (2.01) | Mean (SD) after strip removal | 44.74 (25.24) | 43.10 (21.71) | 60.00 (21.65) |
| LS mean after strip removal | 0.60 | -0.25 (-1.19 to 0.69); *P*=0.5946 | 1.10 (0.13 to 2.07); *P*=0.0277 | LS mean change after vs before strip removal (95% CI);  *P* value | -3.40 (-11.2 to 4.37); *P*=0.3838 | -14.90 (-22.1 to -7.68); *P*=0.0001 | 0.34 (-7.17 to 7.85); *P*=0.9287 |
| *P* value for comparison with placebo | -- | *P*=0.2021 | *P*=0.4005 | *P* value for comparison with placebo | -- | *P*=0.0259^d^ | *P*=0.4683 |
| *P* value for comparison with BRNS | -- | -- | *P*=0.0381^d^ | *P* value for comparison with BRNS | -- | -- | *P*=0.0029^d^ |
| **Day 14** |  |  |  |  |  |  |  |
| Mean (SD) before strip removal | NA | NA | NA | Mean (SD) before strip removal | 48.95 (26.51) | 67.80 (24.82) | 70.61 (22.08) |
| Mean (SD) after strip removal | 0.60 (1.54) | -0.45 (2.28) | 0.94 (2.26) | Mean (SD) after strip removal | 45.26 (25.47) | 51.35 (25.20) | 61.11 (21.98) |
| LS mean after strip removal | 0.54 | -0.52 (-1.50 to 0.45); *P*=0.2894 | 0.87 (-0.17 to 1.90); *P*=0.0990 | LS mean change after vs before strip removal (95% CI);  *P* value | -7.66 (-16.8 to 1.49) *P*=0.0990 | -14.82 (-23.1 to -6.53); *P*=0.0007 | -7.03 (-15.9 to 1.79); *P*=0.1157 |
| *P* value for comparison with placebo | -- | *P*=0.1091 | *P*=0.6333 | *P* value for comparison with placebo | -- | *P*=0.2313 | *P*=0.9183 |
| *P* value for comparison with BRNS | -- | -- | *P*=0.0434^d^ | *P* value for comparison with BRNS | -- | -- | *P*=0.1764 |

*BRNS* Breathe Right Nasal Strip, *CI* confidence interval, *ITT* intent-to-treat, *LS* least square, *NA* not available, *SD* standard deviation, *VAS* visual analog scale

^a^Upon removal of the strip, the nose is expected to return to its normal shape; therefore, a return of symptoms (ie, worsening in the categorical and VAS ratings) after the device is removed supports a therapeutic effect of the device

^b^Subjects rated how breathing felt after they removed the strip, using a scale of -5=much worse, 0=same, and 5=much better

^c^VAS scale of 0=extremely difficult to breathe to 100=extremely easy to breathe

^d^Significant difference between treatments
